# Fostering a just culture in healthcare organizations: experiences in practice

**DOI:** 10.1186/s12913-022-08418-z

**Published:** 2022-08-13

**Authors:** Eva van Baarle, Laura Hartman, Sven Rooijakkers, Iris Wallenburg, Jan-Willem Weenink, Roland Bal, Guy Widdershoven

**Affiliations:** 1 Netherlands Defence Academy, Hogeschoollaan 2, 4818 CR Breda, The Netherlands; 2 Amsterdam UMC, Location VUmc, De Boelelaan 1089a, 1081 HV Amsterdam, The Netherlands; 3 Council of Public Health & Society, The Hague, The Netherlands; 4 University of Applied Sciences, Leiden, The Netherlands; 5 Erasmus University, Erasmus School of Health Policy and Management, Rotterdam, The Netherlands; 6 Erasmus University, Erasmus School of Health Policy and Management, Rotterdam, The Netherlands; 7 Erasmus University, Erasmus School of Health Policy and Management, Rotterdam, The Netherlands; 8 Amsterdam UMC, Location VUmc, Amsterdam, The Netherlands

**Keywords:** Just culture, Patient safety, Learning, Openness, Accountability, Emotions, Exemplary behavior

## Abstract

**Background:**

A just culture is regarded as vital for learning from errors and fostering patient safety. Key to a just culture after incidents is a focus on learning rather than blaming. Existing research on just culture is mostly theoretical in nature.

**Aim:**

This study aims to explore requirements and challenges for fostering a just culture within healthcare organizations.

**Methods:**

We examined initiatives to foster the development of a just culture in five healthcare organizations in the Netherlands. Data were collected through interviews with stakeholders and observations of project group meetings in the organizations.

**Results:**

According to healthcare professionals, open communication is particularly important, paying attention to different perspectives on an incident. A challenge related to open communication is how to address individual responsibility and accountability. Next, room for emotions is regarded as crucial. Emotions are related to the direct consequences of incidents, but also to the response of the outside world, including the media and the health inspectorate.

**Conclusions:**

A challenge in relation to emotions is how to combine attention for emotions with focusing on facts, both within and outside the organization. Finally, healthcare professionals attach importance to commitment and exemplary behavior of management. A challenge as a manager here is how to keep distance while also showing commitment. Another challenge is how to combine openness with privacy of the parties involved, and how to deal with less nuanced views in other layers of the organization and in the outside world. Organizing reflection on the experienced tensions may help to find the right balance.

## Background

Learning from errors is an important requirement for all organizations. It commonly denotes reflection on (nearly) incidents that have occurred, in order to understand their causes, and develop new knowledge that can be applied to future decisions and actions [1, 2]. Learning from errors allows organizations to improve safety, reliability, and resilience [3–5]. While ‘compliance strategies’ focus on rules and behavior of individual employees, various authors show that errors involve not only individual factors, but structural and institutional factors as well, and that a combined effort is necessary to foster an ‘ethical climate’ or learning environment that supports ethically sound behavior, and to foster a sense of shared accountability among employees [6–9].

Within healthcare literature, improving patient safety is often related to learning from incidents [10]. The term *just culture* is commonly used to describe processes within organizations aiming to achieve a fair conclusion for those involved in an incident or a near miss. Key to a just culture is a focus on openness, repairing harm, and learning rather than blaming. Researchers such as Leape, [11] Wu, [12] and Dekker, [13] argue that the use of retributive justice mechanisms, focusing on punishment, hinders the ability of an organization to learn from mistakes. They argue that a change is needed towards restorative justice, aiming to repair the damage and restore the well-being of all those involved. Restorative just culture also focuses on fostering a culture in which employees dare to speak up and voice concerns, not only when errors have occurred but more generally to improve healthcare practices [14]. Whilst, as the label suggests, a just culture process is intended to promote a fair conclusion for those involved and a positive organizational culture, there is considerable controversy over whether just culture processes do in fact achieve these goals [15, 16]. Organizational literature shows that fostering safety culture is complex and may fail to reach intended outcomes [17, 18].

The theoretical concept of just culture as both a process and an intended outcome has received considerable attention within safety science literature. Several studies indicate that the use of sanctions damages the ability of an organization to learn and create mutual trust between management and the workforce [19]. Individuals in organizations may be reluctant to report negative information, especially when this can lead to disciplinary sanctions, or result in being blamed because of an error [20]. The fair treatment of professionals supports a culture of openness and learning by making employees feel confident to speak up when things go wrong, rather than fearing blame.

Despite the volume of theoretical literature on just culture, there has been little examination of the experiences in real life practice [10]. This study therefore focuses on experiences with fostering a just culture within healthcare organizations and explores requirements and challenges in practice. We conducted a qualitative study, investigating initiatives to foster the development of a just culture in five healthcare organizations in the Netherlands. Data were collected through interviews with stakeholders and observations of project group meetings in the organizations.

In the following we first provide a description of the setting and initiatives in the five healthcare organizations and the methods used. We then present our findings on requirements and challenges concerning fostering a just culture in practice. We conclude this study with a discussion of our findings and practical implications.

## Method

### Setting

The presented research was undertaken at the request of the Dutch Health and Youth Care Inspectorate (further: inspectorate), to promote a just culture in healthcare organizations. In recent years, the inspectorate has focused its policy from supervision and reports of serious incidents to improving the process of learning from incidents within healthcare organizations.

In preparation of the study, the inspectorate organized a meeting for healthcare organizations interested in encouraging a just culture, which is part of the inspectorate’s broader aim of improving quality of care by stimulating healthcare organizations to invest in learning and reflective methods. Organizations were invited to participate in a pilot to experiment with fostering a just culture. The participating healthcare organizations were stimulated to choose an activity, theme or topic which was most suitable for them. This made sure that the theme of the pilot addressed a need, theme, topic or question that was experienced as problematic in the organization. Five organizations decided to participate: three large out-patient organizations for mental health care (MH) and two hospitals (H). The inspectorate was not involved in the experiments. The participating organizations and the inspectorate met twice in the course of the projects to exchange experiences and discuss lessons learned. In this paper, we particularly focus on the experiments within the organizations.

The qualitative dataset from two healthcare sectors enables us to understand processes of fostering just culture. However, as participating organizations were motivated to work on just culture, these organizations may not be representative for mental healthcare and hospital sectors, they can be viewed as frontrunners. Table 1 provides an overview of the objectives and initiatives to foster a just culture in the participating organizations.

**Table 1:** Description of objectives and initiatives to foster a just culture

Organization	Objectives	Initiatives
MH 1	Strengthening the involvement and shared ownership of employees in accident investigations and learning from incidents, including suicide attempts.	Series of dialogue sessions with employees aimed at exploring past experiences with accident investigations. Participants had various professional backgrounds across teams (managers; psychiatrists; psychologist; psychiatric nurses and psychotherapists). A feedback session in which the findings of these sessions were discussed by means of a world café method with all participating employees.
MH 2	Searching an appropriate way to 1) learn from incidents of sexual boundary transgressions of mental health professionals towards patients, and 2) prevent future sexual boundaries transgressions.	Dialogue sessions on sexuality and sexual boundary violations including a session with two former patients (victims) and team members discussing the incidents and focus on what the team and the organization could learn from this incident in order to prevent future sexual boundary violations.
MH 3	Evaluation the organization’s patient safety policy through a Just Culture perspective.	The researchers observed and conducted interviews about the extensive policy on patient safety that the organization had implemented. For instance a safety café, in which people can talk freely about fallibility and how safety within healthcare can be improved. During the safety café, people shared personal experiences with regard to incidents.
H 1	Improving internal adverse events investigations based.	Two workshops were organized for incident-investigators within the organization. Both workshops aimed at fostering reflection and learning from current research strategies.
H 2	Developing an approach to quality of care based on “learning from what goes well” and “personal involvement”.	Weekly quality-meetings to complement existing complication-meetings. During these meetings discharged patients are discussed as well as scheduled admissions and operations.

### Data collection

Data were collected through interviews and focus groups with stakeholders and observations of project group meetings in the organizations. Table 2 provides an overview of data sources.

**Table 2:** Overview of data sources

Organization	Data collection	Researchers
MH 1	7 dialogue sessions, each 60 minutes with an average of 4 participants from different layers of the organization 1 feedback-meeting	EvB
MH 2	10 interviews dialogue session participants 2 dialogue sessions, each 120 minutes 6 interviews with participants 2 interviews with members of the management board	EvB, JW
MH 3	17 interviews 4 observations 2 focus groups 1 feedback-meeting	LH, SR
H 1	11 interviews with 14 participants (some participants were paired) 2 focus groups	LH, SR
H 2	7 interviews 12 observations 1 congress: observations	IW
Meetings with all participating organizations	2 meetings: reporting and observations	EvB, NK, JW, LH, SR, RB, GW

### Data analysis

The analysis focused on recognizing and mapping patterns in the experiences of the participants. In the analysis of the transcripts and notes of the interviews, focus groups and observations, researchers focused on “identifying, analyzing, and reporting patterns (themes and subthemes)” [21]. Starting with the first interview, researchers aimed to inductively build an overview of these patterns through open coding. New interviews were used to check and validate already recognized themes, and add new themes as they emerged, resulting in saturation. Findings from the transcripts, observations and meetings in each organization were discussed within the research group and fed back to participating organizations throughout the project. A collaborative discussion in the research group was organized after the open coding, resulting in more general themes, guided by the research questions.

### Ethical considerations

All participants were informed about the study and gave approval for the audio recording. They were informed about how the data would be analyzed, how access to the data was organized and how anonymity was guaranteed. In order to guarantee anonymity, some details from quotes are adjusted. Moreover, it is not indicated which quote belongs to which organization. All methods were performed in accordance with the relevant guidelines and regulations. The study was examined by the Ethical Review Board of Erasmus MC (MEC-2018-054).

## Results

Data analysis reveals three main themes which are important for fostering a just culture in healthcare organizations: A. Open communication, B. Room for emotions, and C. Involvement of managers. For each theme, we will present requirements and challenges.

A. Open communication

Participants argue that in order to foster a just culture, open communication is vital. It is important to support participants to postpone their judgements, to create space for different perspectives, to prevent pointing out individuals who are ‘to blame’, and jointly examine underlying structural and cultural aspects.

*What I really appreciate in these situations, when something goes wrong, and we have a team meeting using the intervision method, that we can really discuss it. It is precisely at such moments that we take time to really listen to one another. In our daily routines we are always in a hurry. These intervision moments provide us with time to reflect on the situation, to learn as a team. Not to focus on what you can do as an individual, but on what we can do as a team in such a situation.*

According to participants, organized moments for dialogue, such as intervision (peer-to-peer coaching) meetings, can contribute to a joint reflection on incidents, aimed at understanding underlying structural and cultural causes, and learning from that. This joint reflection is experienced as an expression of a shared responsibility. Other methods participants refer to are: organized feedback, incident and quality meetings (also focusing on what went well and why), dialogue-sessions, reflection meetings, safety cafes and moral case deliberation. In all these methods, postponing one’s judgement and adopting a listening attitude is key. As one of the participants stated, reviewing working processes has significant added value and should happen more often:

*I think this [moral case deliberation] is a way to enhance our joint sense of responsibility. In teams and departments, we could more often reflect on processes: how do we work and who are responsible? Bringing together all these different perspectives with regard to the same processes.*

A crucial element of open communication is preventing to put the blame on individual professionals. Yet, some participants feel that this may make it difficult to ask others to take responsibility and be accountable for their behavior.

*Not that I would like to say that or that per person, but there were signals and many colleagues were actually aware of that.*

Another challenge is that openness may have consequences beyond the specific setting, since incidents result in responses on various layers, both within and outside the institution. Being open about one’s contribution to an incident may result in becoming the object of an investigation by the inspectorate.

*You realize that the report could be sent to the inspectorate, they may be looking from another perspective and also you have a medical court breathing down your neck. We are talking about just culture, but how open can we really communicate if in the background there is always a Damocles sword above our heads?*

Whereas openness is thus seen as fundamental for learning, it also creates tensions, both within the team to hold team members accountable, and towards the outside world where incidents might be taken up in a less just manner and may even have far-reaching judicial consequences.

B. Room for emotions

Healthcare professionals work in emotionally charged settings, including for instance incidents involving aggressive behavior, suicide attempts, sexual boundary violations and serious or even fatal medical errors. Participants report the emotional impact of incidents on themselves and on the team as a consequence:

*It gets to your head, there are colleagues who have suffered damage, occupational trauma so to say, they have become very cautious. It’s hard to rebuild confidence and take steps towards these kind of responsibilities, it is a slow process.*

Not leaving room for emotions can have serious impact on individuals and teams. In the participating organization where incidents of sexual boundary transgressions had taken place a respondent mentioned the impact of paying insufficient attention to the emotional impact of these transgressions on colleagues:

*What I found really shocking in this process, is that the employees, well, those involved in these incidents, they burst into tears in these sessions. And if after two years you still... if you’re still so sensitive, it means you haven’t found the space [to deal with it] all through these two years. You haven’t talked about it with others for two whole years.*

Emotions are related to the direct consequences of incidents, but also to the response of the outside world, including the media and the inspectorate. Rather than viewing emotions as barriers to rationality and investigation activities, discussing incidents including their emotional impact can contribute to learning processes and subsequently help foster a just culture in healthcare organizations.

A challenge for paying attention to emotions is that investigative bodies, both within and outside of the organization, focus on facts.

*The commission aims at collecting relevant facts, I understand that. Members of the committee are sitting there with this ‘neutral’ attitude and everything is written down very carefully. There is no emotional feedback whatsoever, I do miss that.*

In sum, attention for emotions and examining emotions appears to contribute to fostering a just culture. However, our findings show that is challenging to do so, as actors within and outside the organization are focused on fact-finding.

III. Involvement of management

According to participants, the involvement of management is vital to fostering a just culture. By management we refer to a variety of hierarchy in an organization: a team leader in relation to the team, a medical director in relation to psychiatrists, a medical specialist in relation to nurses, a board member in relation to heads of departments, etc. Management can accommodate open conversations, intervision, feedback moments and contribute to a department culture characterized by open communication. Management can also show exemplary behavior.

*I have experienced that a medical specialist who had a fantastic career as lecturer as well, underwent an incident in his last months. He told all his associates [openly about it], and if someone in his position tells such stories, you know you are in a just culture.*

Regular informal conversations, as well as showing concern for employees and being vulnerable in doing so, is experienced by participants as contributing to a feeling of safety and trust, fostering collective learning processes. According to the participants, management should pay attention to the potential impact of calamities or incidents on employees.

*You don’t make a mistake on purpose, and if you do make a mistake, you are probably the first to acknowledge this, it’s a really upsetting experience. It doesn’t need to be rubbed in your face.*

Yet, participants also state that management can be too close to employees and team processes, which can make it more difficult to critically address behavior:

*I tend to keep some distance to the team members, and perhaps even more after what happened, so I do not engage in friendships. In a former team I was sucked into loyalties.*

Open communication by management can furthermore be challenged by privacy rules aimed at protecting individual employees.

*We had this silence protocol, we were not allowed to say anything. You have a lot of information and the team asks “what is this mess”, and ends up being angry with you. It takes forever. I found that a very, a very difficult period …*. *You have the information but are not allowed to speak.*

Open communication by management can also be challenged by views in higher layers in the organization or the outside world. These higher levels appear to be more sensitive to the outside world, such as the media and the inspectorate, and in an attempt to protect the reputation of organizations after incidents this can draw attention to individual accountability, blame.

*If we would lock ourselves in a cage, that would provide more insight, but findings should not be written down or end up in the media.*

Participants argue that involvement of management fosters a just culture. Yet, management should not become too close. Procedures aimed at protecting privacy are experienced as barriers to open communication in the team. Also, and in contrast to the aims of learning, the top of the organization or the outside world urges to identify ‘the guilty party’. In case of serious incidents, there is little attention for taking into account the broader context of an incident. Also, nuanced outcomes of internal reflections can be easily misunderstood.

## Discussion and conclusions

Open communication, including space to openly discuss incidents and emotions is perceived as important to foster a just culture. A challenge related to open communication is how to address individual responsibility and accountability. Another challenge is how to take into account consequences of openness for procedures. Next, room for emotions is regarded as crucial, as the emotional impact of incidents on individuals as well as teams is easily underestimated. Discussing incidents including their emotional impact on an individual and team level may contribute to learning processes and subsequently help foster a just culture [12, 22]. A challenge is how to address emotions in the context of a focus on facts and an outside world that wants to figure out who has been guilty. Finally, participants attach importance to commitment and exemplary behavior of management. A challenge here is how as a manager to keep enough distance while also showing commitment to the team. Another challenge is how to deal with privacy rules and perceptions regarding external actors.

Our results illustrate the complexity, challenges and tensions of working on fostering just culture in practice. Below, we focus on the identified tensions between openness and accountability, and on tensions between different layers within the organization as well between the organization and the outside world.

First, the tension between openness and accountability. This tension is also visible in the literature, as Reason and Marx argue for a balance between blame, which may result in a lack of learning, [3, 11–13, 23–25] and no-blame, which may promote a lack of accountability [26]. Our results show that by some participants an open dialogue was perceived as never pointing at individual accountability. However, promoting safety through just culture is not a simple call for ‘no blame’ – but rather taking into account that accountability can be both retrospective and prospective [13, 27]. Restorative justice aims to achieve accountability by listening to multiple accounts and looking ahead at what must be done to repair the trust and relationships that were harmed [16]. By doing so it allows room for collective accountability in complex organizations which often depend on collaborative work. This does not exclude retrospective accountability of individual persons. After involving and listening to all involved parties taking individual measures remains a possible outcome. Particularly in case of serious incidents, for instance concerning sexual boundary transgressions, retrospective individual accountability can be combined with prospective collaborative responsibility.

Second, our results indicate that working towards a just culture is a multi-layered process, as relationships in and between different layers within the organization, and with the outside world, play a major role [28]. The higher levels in the organization, being more sensitive to the outside world, such as the media and the inspectorate, draw attention to individual accountability, blame and retribution rather than on a restorative just culture — especially when it comes to calamities. Applying the principles of a restorative just culture demands action, courage and responsibility from management, especially when there is internal or external pressure for blaming and retributive sanctions.

How to deal with the identified tensions? Our results point towards the importance of postponing judgement and a listening attitude. Organized reflection methods and open communication, not only on incidents, but also on tensions and dilemmas in practice, can be helpful [29]. The inspectorate could also adopt regulatory procedures that support reflection and learning in organizations. An example of a structured reflection method is Moral Case Deliberation [30]. By focusing on various values involved in a dilemma in a concrete situation, a wider perspective of the situation can be developed, and participants learn jointly how to find an answer which does justice to these values. In that way, it is possible to find a balance between for example openness and accountability, attention for emotions and examining facts, and between team learning and taking into account the external reputation of the organization. We conclude that for fostering a just culture, not only reflection on the incidents themselves, but also reflection on the tensions involved in learning from incidents is of crucial importance.

## Data Availability

The datasets generated and analyzed during the current study are not publicly available due to the authors not having obtained consent from the participants to share the full interview or dialogue session transcripts. The datasets are available from the corresponding author on reasonable request and with permission of the interviewees.
